# Reconstruction of the insulin-like signalling pathway of *Haemonchus contortus*

**DOI:** 10.1186/s13071-016-1341-8

**Published:** 2016-02-03

**Authors:** Namitha Mohandas, Min Hu, Andreas J. Stroehlein, Neil D. Young, Paul W. Sternberg, James B. Lok, Robin B. Gasser

**Affiliations:** The University of Melbourne, Faculty of Veterinary and Agricultural Sciences, Parkville, VIC Australia; State Key Laboratory of Agricultural Microbiology, College of Veterinary Medicine, Huazhong Agricultural University, Wuhan, 430070 Hubei China; HHMI, Division of Biology, California Institute of Technology, Pasadena, CA USA; Department of Pathobiology, School of Veterinary Medicine, University of Pennsylvania, 3800 Spruce Street, Philadelphia, PA 19104 USA

**Keywords:** Nematode, *Haemonchus contortus*, Insulin-like signalling pathway

## Abstract

**Background:**

In the present study, we reconstructed the insulin/insulin-like growth factor 1 signalling (IIS) pathway for *Haemonchus contortus*, which is one of the most important eukaryotic pathogens of livestock worldwide and is related to the free-living nematode *Caenorhabditis elegans*.

**Methods:**

We curated full-length open-reading frames from assembled transcripts, defined the complement of genes that encode proteins involved in this pathway and then investigated the transcription profiles of these genes for all key developmental stages of *H. contortus*.

**Results:**

The core components of the IIS pathway are similar to their respective homologs in *C. elegans*. However, there is considerable variation in the numbers of isoforms between *H. contortus* and *C. elegans* and an absence of AKT-2 and DDL-2 homologs from *H. contortus*. Interestingly, DAF-16 has a single isoform in *H. contortus* compared with 12 in *C. elegans*, suggesting novel functional roles in the parasitic nematode. Some IIS proteins, such as DAF-18 and SGK-1, vary in their functional domains, indicating distinct roles from their homologs in *C. elegans*.

**Conclusions:**

This study paves the way for the further characterization of key signalling pathways in other socioeconomically important parasites and should help understand the complex mechanisms involved in developmental processes.

**Electronic supplementary material:**

The online version of this article (doi:10.1186/s13071-016-1341-8) contains supplementary material, which is available to authorized users.

## Background

Roundworms (nematodes) are one of the most diverse groups of organisms on the planet. Some are free-living, and many are parasitic, causing substantial disease and socioeconomic problems globally. For example, *Haemonchus contortus* (the barber’s pole worm; order Strongylida) is one of the most destructive parasitic nematodes of livestock animals (small ruminants, including sheep and goats) due to its high pathogenicity and widespread occurrence around the world [1]. This nematode feeds on blood from capillaries in the stomach (abomasum) wall, and causes haemorrhagic gastritis, anaemia, oedema and associated complications, often leading to the death of severely affected animals. *H. contortus* is transmitted orally from contaminated pasture to the host through a direct life cycle involving three free-living larval stages, of which the infective third larval stage (iL3) is ingested [2]. After a histotropic phase in the host animal, the larvae develop to the fourth stage (L4) and then to adults, which both feed on blood and cause pathogenic effects in the host animal.

The recent characterization of transcriptomes and draft genomes of *H. contortus* [3, 4] provides a solid basis for future studies of its developmental and reproductive biology using genetic, genomic, proteomic and metabolomic tools. However, a lack of tractable functional genomic tools for *H. contortus* and related parasitic nematodes, and an inability to maintain their complete life cycles in vitro, hampers functional investigations of genes and gene products in these nematodes (cf. [5–12]). This contrasts the situation for the free-living nematode, *Caenorhabditis elegans*, the best characterized metazoan organism, which can be readily maintained, and used to investigate fundamental processes and mechanisms, such as dauer formation [13].

*Caenorhabditis elegans*, which belongs to “clade V” [14], is relatively closely related to *H. contortus*. Published information [7, 8, 15–17] indicates similarity in entry into and exit from the “dauer state” between *C. elegans* and strongylid nematodes [18]. This arrested state occurs in *C. elegans* when the nematode encounters harsh environmental conditions, such as starvation, crowding and/or a high temperature [19, 20]. The dauer form can survive for several months and then resume development to reproductive adults when conditions improve [19]. Consistent with *C. elegans*, *H. contortus* and related nematodes have a similar third larval stage (L3), which is relatively resistant to unfavourable conditions and does not feed because it is encased by a cuticular sheath [2]. The “dauer hypothesis” [18] contends that the resumption of iL3 development in parasitic nematodes is functionally and developmentally analogous to the exit from dauer in *C. elegans*, and is regulated by similar mechanisms [13, 15, 16, 21].

Dauer development is governed by multiple signalling pathways, including the insulin/insulin-like growth factor 1 (IGF1)-like signalling (IIS) pathway [13], which, in *C. elegans*, comprises proteins such as DAF-2 (insulin-like receptor kinase [22–24]), AGE-1 (phosphoinositide-3 (PI3) kinase [25–27]) and DAF-16 (FOXO-class transcription factor [28–30]). In *C. elegans*, signalling via DAF-2 activates AKT-1/2 by phosphorylation which, in turn, negatively regulates DAF-16, which functions as a central mediator of multiple biological processes, such as growth, development, reproduction, longevity, age and stress resistance [31].

While much is known about the IIS pathway in *C. elegans* (reviewed in [31]), only a few studies have explored the functions of selected parts of this pathway in *H. contortus* [32–34], and no study has yet investigated its full composition in this parasitic nematode. Therefore, in the present study, we (i) curated the full-length open reading frames (ORFs) and defined the complement of genes that encode peptides/proteins involved in IIS, (ii) studied the interactions of these genes and (iii) examined their transcription profiles in all key developmental stages of *H. contortus*.

## Methods

We employed data relating to a published draft genome as well as transcriptomes of all key developmental stages (egg, first- to fourth-stage larvae (L1, L2, L3 and L4) and adult) and both sexes (L4 and adult) of *H. contortus* (NCBI BioProject accession no. PRJNA205202; [4, 35]). This draft genome is **~** 320 Mb in size and has been predicted to encode 23,610 proteins [4].

### Identification of genomic scaffolds containing genes encoding IIS pathway components

From the complete, assembled transcriptome representing all eight stages or sexes of *H. contortus* [4], we identified and extracted assembled transcripts based on their homology matches (*E*-value cut-off: 10^−5^) to all genes encoding insulin/insulin-like growth factor 1 signalling (IIS) proteins in *C. elegans* [35]*.* Then, we identified genomic scaffolds containing regions of homology to known IIS genes by mapping (*E*-value cut-off: 10^−5^) all assembled transcripts using BLAT [36]. We also used IIS genes from the *H. contortus* draft genome predicted previously using MAKER2 [4, 37]. Open reading frames (ORFs) of individual assembled transcripts were inferred using the program GeneMark-ES [38, 39]. Using the Integrative Genomics Viewer (IGV) [40, 41], we then visually integrated all of these data to obtain a consensus sequence for individual coding regions.

### Identification of protein domains, families and subfamilies

Identifying IIS protein genes encoded in the draft genome allowed us to then define the complete set of full-length transcripts. ORFs were verified and corresponding coding regions inferred from these full-length transcripts using ORF-finder [42]. Each predicted protein was characterized by its primary amino acid sequence and structural and/or functional domains, inferred using all databases (i.e., PROSITE, HAMAP, Pfam, PRINTS, ProDom, SMART, TIGRFAMs, PIRSF, SUPERFAMILY, CATH-Gene3D and PANTHER) within InterProScan v.5.14.53 [43, 44]. Individual predicted proteins were classified according to family and/or subfamily using information in the PANTHER database v.9.0 [45, 46]. Following a comparison of those inferred from transcripts and genomic exons encoding IIS genes with the corresponding *C. elegans* homologs, we were able to infer the full complement of full-length transcripts and protein sequences.

### Analysis of differential transcription

Each set of quality-filtered, paired-end RNA-seq reads for each individual developmental stage or sex of *H. contortus* was mapped to the final complement of full-length IIS transcripts using Burrows-Wheeler Aligner (BWA) software [47]. For each stage/sex, the numbers of reads that mapped to individual transcripts were established using the SAM tools algorithm [48]. The resultant read counts per transcript per developmental stage were used as input data for DESeq2 and edgeR [49, 50]. Differential transcription was calculated by pairwise comparison of all free-living (egg, L1, L2 and L3) and parasitic (L4 and adult) stages. Genes were recorded as differentially transcribed, using edgeR-calculated common and gene-wise dispersion factors, if the log_2_ fold change (log_2_ FC) between free-living and parasitic stages compared with the normalised read count data was ≥ 2, with a false discovery rate (FDR) of ≤ 0.05. A heat map (representing mapped reads) was produced using the heatmap.2 function in the gplots package in R [51].

## Results

### Identification and characterization of IIS signalling protein genes

From the complete, assembled transcriptome of *H. contortus*, we identified and extracted 3792 assembled transcripts based on their homology matches (*E*-value cut-off: 10^−5^) to the 45 IIS protein genes, and then located the regions in genomic scaffolds to which these assembled transcripts mapped. The manual curation of the matching genomic and transcriptomic data for *H. contortus* identified 27 of 41 IIS gene homologs, and 4 of 40 insulin-like peptide (ILP) gene homologs using *C. elegans* genes as references.

Four, 18 and 9 of the 31 full-length transcripts encoded proteins involved in the upstream, conserved and downstream components of the IIS pathway, respectively. The features of these 31 predicted proteins (including lengths and pairwise sequence identities to their *C. elegans* homologs) are summarized in Table 1. Specifically, the numbers of isoforms of the predicted IIS genes varied from those of homologs encoded in *C. elegans*. Genes, such as the insulin-like peptide (*ins-1*), phosphoinositide 3-kinase (*age-1/pi3k*), heat-shock factor (*hsf-1*) and serine/threonine phosphatase (*pptr-1*), each have two or three isoforms in *H. contortus,* compared with only one molecule in *C. elegans*. In contrast, genes, such as those encoding the insulin receptor (*daf-2*), the phosphoinositide-dependent kinase (*pdk-1*), the 14-3-3 protein (*par-5* and *ftt-2*), the Nrf family transcription factor (*skn-1*) and the FoxO family transcription factor (*daf-16*), express single transcripts in *H. contortus* compared with 2 to 12 isoforms in *C. elegans* (Table 1). Individual predicted protein sequences ranged in length from 70 to 1455 amino acids, comparable with their corresponding *C. elegans* homologs, and these sequences shared 14.9 % to 84.3 % identity to their *C. elegans* homologs upon pairwise comparison (Table 1).

**Table 1:** Pairwise comparisons of sequence identity (%) of proteins representing the insulin/insulin-like growth factor 1(IGF1)-like signalling pathway (IIS) between *Haemonchus contortus* and *Caenorhabditis elegans*

Protein	Scaffold	Transcripts representing the IIS genes	Length (aa)	Homologs in *C. elegans* (Gene code)	Length (aa)	Pairwise sequence identity (%)
*Hc*-INS-1a	scaffold1633	Locus_6343_Transcript_1/2_Confidence_0.667_Length_624	100	F13B12.5	109	42.0
*Hc*-INS-1b	scaffold11919	Locus_3137_Transcript_1/2_Confidence_0.750_Length_686	100	F13B12.5	109	42.0
*Hc*-INS-17	-	Locus_4104_Transcript_1/1_Confidence_1.000_Length_511	106	F56F3.6	108	54.7
*Hc*-INS-18	-	Locus_1267_Transcript_2/3_Confidence_0.600_Length_720	70	T28B8.2	95	47.4
*Hc*-DAF-2	scaffold13413	Locus_7014_Transcript_2/3_Confidence_0.714_Length_5695	1455	Y55D5A.5a, b, c*, d, e, f, g	672–1928	25.5–34.6
*Hc*-IST-1	scaffold434	Locus_5794_Transcript_4/8_Confidence_0.650_Length_4263 + Locus_5794_Transcript_6/8_Confidence_0.500_Length_3032	1441	C54D1.3	1003	18.4
*Hc*-AAP-1	scaffold6618	Locus_7479_Transcript_1/1_Confidence_1.000_Length_1541	427	Y110A7A.10	522	36.1
*Hc*-AGE-1a	C279481	Locus_4139_Transcript_1/1_Confidence_1.000_Length_3713	1156	B0334.8a	1182	40.5
*Hc*-AGE-1b	scaffold1062	Locus_10568_Transcript_2/2_Confidence_0.800_Length_3699	1149	B0334.8a	1182	40.6
*Hc*-DAF-18		Locus_6617_Transcript_3/3_Confidence_0.714_Length_3487	800	T07A9.6	962	24.0
*Hc*-PDK-1	C457723	Locus_2599_Transcript_2/3_Confidence_0.500_Length_2764	576	H42K12.1a, b*	632–636	41.8–42.4
*Hc*-SGK-1a	-	Locus_1010_Transcript_1/1_Confidence_1.000_Length_1434	462	W10G6.2a*, b	453–463	60.0–61.2
*Hc*-SGK-1b	-	Locus_5956_Transcript_1/1_Confidence_1.000_Length_1384	428	W10G6.2a*, b*	453–463	63.1
*Hc*-AKT-1a	scaffold15637	Locus_6593_Transcript_2/2_Confidence_0.857_Length_2281	540	C12D8.10a*, b, c	254–546	73.2–75.9
*Hc*-AKT-1b	scaffold17120	Locus_44_Transcript_10/11_Confidence_0.651_Length_6534	552	C12D8.10a, b*, c	254–546	73.2–77.6
*Hc*-PPTR-1a	scaffold4564	Locus_1388_Transcript_2/4_Confidence_0.700_Length_4420	542	W08G11	542	80.1
*Hc*-PPTR-1b	C217263	Locus_3418_Transcript_2/2_Confidence_0.667_Length_3679	542	W08G11	542	80.1
*Hc*-PPTR-1c	C426137	Locus_351_Transcript_5/7_Confidence_0.556_Length_5020	542	W08G11	542	80.1
*Hc*-PPTR-2	scaffold6183	Locus_8402_Transcript_1/1_Confidence_1.000_Length_3588	476	C13G3.3a*, b, c*, d*	557–607	70.1–71.2
*Hc*-FTT-2	-	Locus_4342_Transcript_1/6_Confidence_0.556_Length_1420	206	F52D10.3a, b*	198–248	80.5–84.3
*Hc*-PAR-5	-	Locus_4342_Transcript_5/6_Confidence_0.667_Length_1898 + Locus_4342_Transcript_3/6_Confidence_0.667_Length_1669	201	M117.2a*, b	126–248	48.4–82.6
*Hc*-DAF-16	C472265	Locus_5600_Transcript_3/3_Confidence_0.714_Length_3611	589	R13H8.1a, b, c, d, e, f, g, h, i, k, l, m*	303–589	32.4–43.2
*Hc*-SKN-1	scaffold10742	Locus_3183_Transcript_5/6_Confidence_0.684_Length_3607	612	T19E7.2a, b, c, d*	223–623	30.3–38.5
*Hc*-HSF-1a	scaffold1518	Locus_7749_Transcript_2/2_Confidence_0.875_Length_2372	544	Y53C10A.12	671	33.5
*Hc*-HSF-1b	scaffold3772	Locus_497_Transcript_5/5_Confidence_0.577_Length_2356	544	Y53C10A.12	671	33.5
*Hc*-HSB-1	scaffold9500	Locus_3235_Transcript_1/1_Confidence_1.000_Length_453	91	K08E7.2	80	60.0
*Hc*-DDL-1	scaffold11860	Locus_4675_Transcript_1/2_Confidence_0.750_Length_767 + Locus_8707_Transcript_2/3_Confidence_0.667_Length_738	129	F59E12.10	189	27.1
*Hc*-EGL-9a	scaffold12571	Locus_5190_Transcript_1/1_Confidence_1.000_Length_1701	504	F22E12.4a, b, c, d*, e	363–723	14.9–48.6
*Hc*-EGL-9b	C449377	Locus_3254_Transcript_2/2_Confidence_0.333_Length_2254	482	F22E12.4a, b, c, d*, e	363–723	14.9–51.2
*Hc*-EGL-9c	scaffold12558	Locus_1766_Transcript_1/1_Confidence_1.000_Length_2016	482	F22E12.4a, b, c*, d, e	363–723	14.9–49.5
*Hc*-EGL-9d	C457287	Locus_4349_Transcript_1/1_Confidence_1.000_Length_1791	482	F22E12.4a, b, c*, d, e	363–723	14.9–49.5

*C. elegans* isoforms with the highest pairwise sequence identity to *H. contortus* homologs are marked (*)

InterProScan analysis allowed the classification of predicted IIS proteins, based on domains and protein signatures (Additional file 1: Figure S1). The proteins predicted for *H. contortus* (i.e., *Hc-*PDK-1, −PPTR-1, −AKT-1, −DAF-16, −HSB-1, −SKN-1 and *-*EGL-9) had the domains and signatures that were consistent with their respective *C. elegans* homologs. *Hc-*DAF-2 consisted of a protein kinase ATP-binding region signature (PS00107), an iron-sulfur binding domain (PS51379) and a furin-like cysteine-rich region (SM00261), which have been found in a number of eukaryotic proteins, such as epidermal growth factor receptor, endoprotease-4 and receptor tyrosine-protein kinase (LET-23), known to be involved in signal transduction by receptor tyrosine kinases [52–55]. *Hc-*DAF-18 lacked the protein tyrosine phosphatase catalytic domain (PTPc) motif and, instead, contained a dual-specificity phosphatase catalytic domain (PF00782). The pleckstrin homology (PH) domain (SM00233) was not present in *Hc*-PDK-1. *Hc*-PPTR-2 (protein phosphatase 2A) consisted of a unique leucine-rich repeat variant (G3DSA: 1.25.10.10) that represents a super-helical structure predicted to aid the binding of large substrates [56]. In comparison to the 14-3-3 proteins FTT-2 and PAR-5 of *C. elegans*, which have two protein signatures in PROSITE, the predicted *H. contortus* proteins have only protein signature 1 (PS00796). PANTHER family and subfamily classifications could not be assigned to some predicted IIS proteins of *H. contortus*, such as *Hc-*INS-1, *Hc-*DDL-1 and *Hc-*PAR-5. The regulatory subunit, *Hc-*IST-1, which contains the PH domain (SM00233; PS50003) belonging to the PH domain-like superfamily (SSF50729) is not present in *C. elegans*. The other adaptor protein, *Hc-*AAP-1, also contains the SH2 (Src homology 2) domain (PR00401), which is a 5-element fingerprint, and corresponds to the core structural element of the protein. The predicted *Hc-*SGK-1 protein consists of the PX (phox) domain (PS50195; SSF64268), which is an important phosphoinositide-binding module with variable lipid-binding specificity [57, 58].

### Transcription profiles

**Fig. 1:**
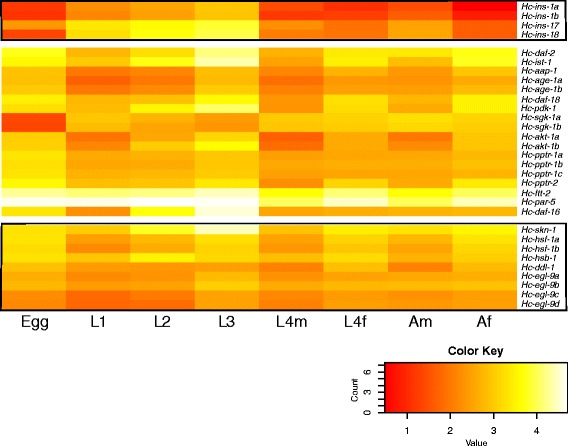
Heat-map displaying transcription profiles for genes (cf. Table 1) involved in insulin/insulin-like growth factor 1 (IGF1)-like signalling (IIS) in *Haemonchus contortus. Hc-ins-1a/1b*, −*ins-17* and *-ins-18* are genes that act upstream (U) of the IIS pathway. Genes *Hc-daf-2*, −*ist-1*, −*aap-1*, −*age-1a/1b*, −*daf-18*, −*pdk-1*, −*sgk-1a/1b*, −*akt-1a/1b*, −*pptr-1a/1b/1c*, −*pptr-2*, −*ftt-2*, −*par-5* and *Hc-daf-16* are part of the core (C) IIS pathway and genes *Hc-skn-1*, −*hsf-1a/1b*, −*hsb-1*, −*ddl-1* and *Hc-egl-9a/b/c/d* are activated downstream (D) of the IIS pathway. Transcription levels in different developmental stages (egg, L1, L2, L3, L4 and A = adult; f = female; m = male) of *H. contortus* (see colour scale): low (red), medium (orange), high (yellow) and very high (white)

Significant differences in transcription were recorded among eight stages/sexes of *H. contortus* for some of the 31 genes involved in the IIS pathway (Fig. 1). The insulin-like peptide genes *Hc-ins-1a*, −*ins-1b*, −*ins-17* and -*ins-18* were highly transcribed in L1 to L3 stages of *H. contortus*, and significantly down-regulated in parasitic stages, particularly female L4 and adult stages. In contrast, downstream genes, such as *Hc-daf-2*, −*aap-1*, −*ist-1, −age-1a*, −*age-1b*, −*daf-18*, −*pdk-1* and *-akt-1*, which encode IIS cytoplasmic signalling proteins, had the highest transcription in eggs, L3s and female adults (Fig. 1), except for *Hc-sgk-1*, which had limited transcription in both egg and L3 stages in comparison to all other stages. The 14-3-3 homologous genes, *Hc-ftt-2* and *Hc-par-5*, known to regulate *daf-16* in *C. elegans* [59, 60] were highly transcribed in all life cycle stages, while *Hc-daf-16* was highly transcribed only in the egg, L2 and L3 stages (Fig. 1). Downstream genes transcriptionally regulated by *daf-16* (*skn-1*, *hsf-1*, *hsb-1*, *ddl-1* and *egl-9*) were transcribed at high levels in eggs, L3s and in females of both haematophagous (i.e., L4 and adult) stages (Fig. 1).

### The IIS signalling pathway of *H. contortus*

Of a total of 81 genes involved in the IIS pathway of *C. elegans* [31], 31 homologs were identified in *H. contortus.* Specifically, in *H. contortus*, four insulin-like peptide (ILP) homologs of 40 in *C. elegans* as well as 9 of 11 downstream components of the IIS pathway were identified. However, *C. elegans akt-2* and *ddl-2* homologs were not found in *H. contortus*, which was confirmed by searching independent genomic and transcriptomic data sets [3]. Therefore, overall, the number of core IIS genes (*Hc-daf-2*, −*ist-1*, −*aap-1*, −*age-1*, −*daf-18*, −*pdk-1*, −*sgk-1*, −*akt-1*, −*pptr-1*, −*ftt-2*, −*par-5* and -*daf-16*) was similar, with variation mainly in the numbers of isoforms between *H. contortus* and *C. elegans*.

**Fig. 2:**
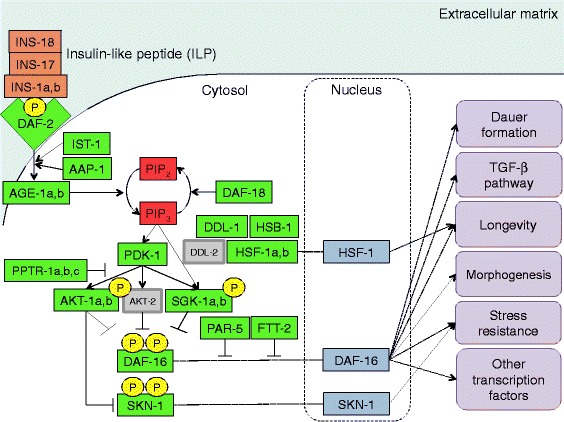
Reconstruction of the insulin/insulin-like growth factor 1 (IGF1)-like signalling (IIS) pathway of *Haemonchus contortus.* Four insulin-like peptides (ILPs) are encoded in *H. contortus* (orange) compared with 40 in *Caenorhabditis elegans*. All core functional gene products (green) of the IIS pathway, except for AKT-2 and DDL-2 (grey), are encoded in *H. contortus*. Additional transcription factors activated downstream of the IIS pathway, such as SKN-1 and HSF-1, are also predicted in *H. contortus*. Phosphatidylinositol (3,4,5)-trisphosphate (PIP_3_) is the product of the class I phosphoinostide 3-kinases (PI 3-kinases) phosphorylation of phosphatidylinositol (4,5)-bisphosphate (PIP_2_); it is a phospholipid in the plasma membrane. Phosphorylation represented by P (yellow). The insulin pathway controls the transcription of various genes, and regulates key processes, including dauer formation, longevity, stress resistance and morphogenesis, as well as other pathways such as TGF-β and involving other key transcription factors

Using information available for *C. elegans* (from WormBase; [35]), we constructed the IIS pathway for the genes predicted in *H. contortus* (Fig. 2). In this pathway, insulin-like peptides encoded by *Hc-ins-1a*, −*ins-1b*, −*ins-17* and -*ins-18* are predicted to interact with DAF-2 insulin receptor, thereby activating the receptor. This, in turn, should facilitate the recruitment of regulatory proteins, *Hc-*IST-1 and *-*AAP-1, to activate phosphoinositide 3-kinase (*Hc-*AGE-1). The activation of *Hc-*AGE-1 likely results in a conversion of phosphatidylinositol 4,5-bisphosphate (PIP_2_) to phosphatidylinositol 4,5-trisphosphate (PIP_3_), which, in turn, activates phosphoinositide-dependent protein kinase-1 (−PDK-1). A regulatory lipid phosphatase, *Hc-*DAF-18, is inferred to act as an antagonist to -AGE-1 activity by dephosphorylating PIP_3_. Downstream serine/threonine kinases, *Hc-*AKT-1 and *Hc-*SGK-1, are predicted to be activated by *Hc-*PDK-1, resulting in the phosphorylation of *Hc-*DAF-16 (FoxO transcription factor). The protein phosphatases *Hc-*PPTR-1 and *-*PPTR-2 act as regulatory subunits that bind and dephosphorylate *Hc-*AKT-1. The phosphorylated *Hc-*DAF-16 interacts with the regulatory proteins *Hc-*FTT-2 and *Hc-*PAR-5, inhibiting its subcellular localization into the nucleus and subsequent regulation of DAF-16 target genes. A number of downstream target genes are involved in various processes, such as stress resistance, dauer formation, the regulation of life span and the activation of transforming growth factor beta (TGF-β) signalling. The regulation of IIS genes is important, as it affects the transcription of downstream gene targets that may result in loss-of-function phenotypes.

## Discussion

Using a bioinformatic approach, we reconstructed the IIS pathway of *Haemonchus contortus* from transcriptomic and genomic data sets for this nematode, and explored the transcription of individual genes as well as their interactions. The composition of the core IIS pathway in *H. contortus* was relatively consistent with that of *C. elegans*, although there were considerable differences between these nematodes in the upstream and downstream targets of this pathway. In the present study, four insulin-like peptides (ILPs) were identified in *H. contortus*, as opposed to 40 ILPs found in *C. elegans* [61–64]. A similar, marked reduction in the repertoire of ILP-encoding transcripts has been observed in the parasitic nematode *Strongyloides stercoralis* (clade IV), where only seven of the 40 ILPs of *C. elegans* are represented [65]. The four ILPs predicted for *H. contortus* all have representative functional domains and protein signatures, including an insulin-like domain representing the insulin/IGF/relaxin family, a disulphide-rich alpha-helical domain and an insulin family signature (cf. Additional file 1: Figure S1). In *C. elegans*, several ILPs (encoded by *ins-1, ins-6, ins-7*) have been shown to regulate dauer formation, longevity and development [31]. Although the functions of all 40 ILPs are not yet known, some (e.g., encoded by *ins-1*, *ins-6*, *ins-7* and *daf-28*) have been extensively studied [63, 64, 66–68]. An interesting feature of these peptides is that they can either act as agonists or antagonists of DAF-2, the only insulin-like receptor kinase in *C. elegans* [63, 66, 67, 69]. While the basis of the differences in function is presently unknown, it is hypothesized that neural inputs from chemosensory neurons in the amphids and transduced by G protein-coupled receptors (GPCRs) therein trigger specific profiles of ILP expression, possibly including optimum levels of agonists and antagonists in one or a few developmental stages, which combine to precisely regulate the insulin signalling pathway response (i.e., downstream gene expression) to the complex set of environmental cues experienced by this free-living nematode [70]. It is likely that ILP profiles are similarly regulated in parasitic nematodes. Indeed, the regulation of ILP expression by upstream cyclic GMP signalling has been observed in *S. stercoralis*, where the administration of 8-bromo-cGMP to cultured larvae elicits naturally occurring profiles of ILP expression, in contrast to the baseline levels of expression observed in untreated controls [71]. The marked reduction in numbers of ILPs in parasitic nematodes examined to date might reflect a rather more specific interaction with their environments relative to more opportunistic free-living organisms, such as *C. elegans*. The homologs of the *C. elegans* ILPs identified in *H. contortus* were encoded by *Hc-ins-1a*, −*ins-1b*, −*ins-17* and *-ins-18*, which all represent antagonists, implying that the regulation of the IIS pathway is dependent on their expression patterns in all life stages of the parasite. High transcription in the L1, L2 and L3 stages of *H. contortus* and considerably lower transcription in L4 and adult stages indicate that the ILPs of *H. contortus* are transcriptionally regulated during the transition from free-living to parasitic stages. By contrast to *H. contortus*, the seven ILPs of *S. stercoralis* constitute a set of peptides whose structures and patterns of expression in free-living and parasitic stages suggest members that are either agonists or antagonists of *Ss*-DAF-2. This apparent diversity of ILP function in *S. stercoralis* could explain the capability of the parasite to undertake either direct development to infective L3s or development to a generation of free-living male and female worms with many biological attributes in common with non-parasitic nematodes.

Some of the intracellular protein components of the IIS pathway showed variation in the functional domains present and in the number of isoforms predicted. For instance, *Hc*-DAF-18 is a homolog of the mammalian PTEN protein, which is a well-recognised phosphatase and a tumour suppressor [72]. In *C. elegans*, DAF-18, a lipid phosphatase, acts by inhibiting the activation of PDK-1 by dephosphorylating phosphatidylinositol (3,4,5)-trisphosphate (PIP_3_) to phosphatidylinositol 4,5-bisphosphate (PIP_2_) by removing the phosphate in the D3 position of the inositol ring [73]. *Ce*-DAF-18 is also suggested to have tyrosine phosphatase activity, based on the presence of its functional catalytic domain. However, in *H. contortus*, while *Hc*-DAF-18 might have the same lipid phosphatase function as its *C. elegans* homolog, the presence of a dual-specificity phosphatase catalytic domain indicates its ability to dephosphorylate both tyrosine- and serine-/threonine-phosphorylated proteins. This suggested, additional function of *Hc*-DAF-18 might hint to a functional role of the signalling pathway in recovery from developmental arrest.

The *C. elegans* insulin-signalling pathway activates two Akt family members, AKT-1 and AKT-2, as well as a serum and glucocorticoid-inducible kinase, SGK-1 downstream of the phosphoinositide-dependent kinase, PDK-1. All of these serine-threonine kinases are activated by AGE-1/PI3K. Previous studies [74–76] have shown that null-mutants of *Ce-akt-1* and *Ce-akt-2* result in non-conditional dauer arrest and an extension of lifespan. A knockdown of *sgk-1* by RNAi also induces an extended lifespan, indicating that it functions in a similar manner to AKT-1 and AKT-2 [77]. Interestingly, in *H. contortus*, only two kinases were predicted downstream of *Hc*-PDK-1, namely *Hc*-AKT-1 and *Hc*-SGK-1. The absence of AKT-2 might indicate a distinct regulation of IIS pathway in this nematode during the switch to the parasitic stage and also during reproduction. The transcription of *Hc-akt-1* was higher in females than in males. The assessment of the functional domains of *Hc*-SGK-1 revealed the presence of a PX (phox) domain, which was not present in its respective *C. elegans* homolog. PX, a phospholipid-binding domain, primarily interacts with PIP_3_ lipids [78]. The presence of this unique domain is likely to compensate for the deficiency of AKT-2 by allowing an activation of *Hc*-SGK-1 by both *Hc*-PDK-1 as well as PIP_3_, thereby effecting the expression of downstream *Hc*-DAF-16 target genes.

DAF-16 is a member of the FoxO family of forkhead transcription factors, which are regulators of growth, metabolism, stress response, cell cycle control and longevity in many organisms [79]. The nuclear translocation of DAF-16 from the cytoplasm is inhibited by the phosphorylation at its RxRxxS/T motifs [80, 81], which are conserved among *C. elegans* DAF-16, mammalian FoxOs and predicted *H. contortusdaf-16*. The *C. elegans* genome encodes twelve DAF-16 transcripts. Although the functions of these isoforms are not known, it is hypothesized that they have distinct tissue distributions in hypodermis, muscle, neurons, and intestine [29, 80–83]. Studies of *Ce*-DAF-16 indicate that biological functions of the isoforms vary according to their tissue distribution [80, 83]. In contrast, in *H. contortus*, a single homolog of DAF-16 was predicted. This finding implies that the array of target genes predicted to be transcriptionally regulated by *Hc*-DAF-16 is controlled by a single homolog, possibly resulting in a more complex regulation of downstream gene targets and distinct phenotypes. Given that the input signals of IIS pathway are also lesser in number compared with *C. elegans*, this information suggests a novel regulatory mechanism that differs between free-living and parasitic nematodes. This hypothesis is bolstered by a similar reduction in transcripts from the *daf-16* ortholog in *S. stercoralis*. Here, there are only two transcripts, designated *Ss-daf-16*a and *Ss-daf-16*b, each expressed under the control of a different promoter [65, 84].

The ‘dauer hypothesis’ suggests that a similar mechanism of action takes place in major signalling pathways including the IIS, cyclic GMP and TGF-β pathways, which control the entry into and exit from arrested development in *C. elegans*, as in parasitic nematodes [18]. The present investigation of genomic and transcriptomic data sets from *H. contortus* suggests that the major intracellular signalling components of IIS, such as *daf-2*, *age-1*, *pdk-1* and *akt-1*, likely have similar functions to *C. elegans* homologs. This hypothesis has been supported for *age-1* orthologs in other parasitic nematodes by the fact that the PI3K inhibitor LY294002 suppresses developmental activation of iL3 under host-like culture conditions [85–87]. However, the roles of key genes encoding ILPs, DAF-18, SGK-1 and DAF-16 in *H. contortus* appear to be distinct.

Although the ‘dauer hypothesis’ usually considers L3 arrest in parasitic nematodes to be analogous to dauer in *C. elegans* [18], *H. contortus* and related nematodes, such as *Ostertagia* and *Cooperia* spp., can undergo hypobiosis at the early L4 stage within the host animal [88]. Given that this latter adaptive state enables transitional parasite survival within the host, and regulates parasite transmission and population size [88], understanding this phenomenon is of critical importance. Hence, future work should evaluate the involvement of IIS and associated signalling pathways in hypobiosis. It would be of particular interest to reconstruct the TGF-β signalling pathway in *H. contortus* and other trichostrongylids, and assess transcription profiles throughout development, given the major contrast in transcription at the L3 stage between various parasitic nematodes including *H. contortus* (up-regulation) and *C. elegans* (down-regulation) (cf. [18]) that suggests an altogether unique function of DAF-7 in parasitic worms. This line of investigation will be interesting in light of the fact that *C. elegans* offers no exact counterpart to the early L4 arrest that occurs in these economically important trichostrongyles. As such, mechanisms of early L4 arrest will likely represent a unique adaptation to parasitism for some clade V nematodes.

## Conclusions

In conclusion, the availability of transcriptomic and draft genomic data sets for *H. contortus* has enabled the first detailed bioinformatic exploration of the IIS pathway in this parasite. We curated the full-length transcripts and defined the complement of genes that encode peptides/proteins involved in this pathway by comparison with *C. elegans*, reconstructed the pathway with these genes and investigated their transcription profiles in key developmental stages of *H. contortus*. We hope that reconstructing the IIS pathway for *H. contortus* will provide a stepping stone for future studies of development, reproduction, ageing, longevity, metabolism and/or behaviour in this important parasitic worm, and a stimulus to explore other signalling pathways in socioeconomically important strongylids.

## Additional file

Additional file 1: Figure S1. A schematic representation of functional domains and motifs as predicted by InterProScan of members of the insulin/insulin-like growth factor 1 (IGF1)-like signalling (IIS) pathway of *Haemonchus contortus* inferred from full-length transcripts and their *Caenorhabditis elegans* homologs. (PDF 216 kb)
