# Supplementary material for: Reconstruction of the insulin-like signalling pathway of Haemonchus contortus
Source: Parasit Vectors. 2016 Feb 3;9:64. doi: 10.1186/s13071-016-1341-8 (PMC4741068; doi:10.1186/s13071-016-1341-8)

Additional file 1: Figure S1 A schematic representation of functional domains and motifs as predicted by InterProScan of members of the insulin/insulin-like growth factor 1 (IGF1)-like signaling (IIS) pathway of *Haemonchus contortus* inferred from full-length transcripts and their *Caenorhabditis elegans* homologs.

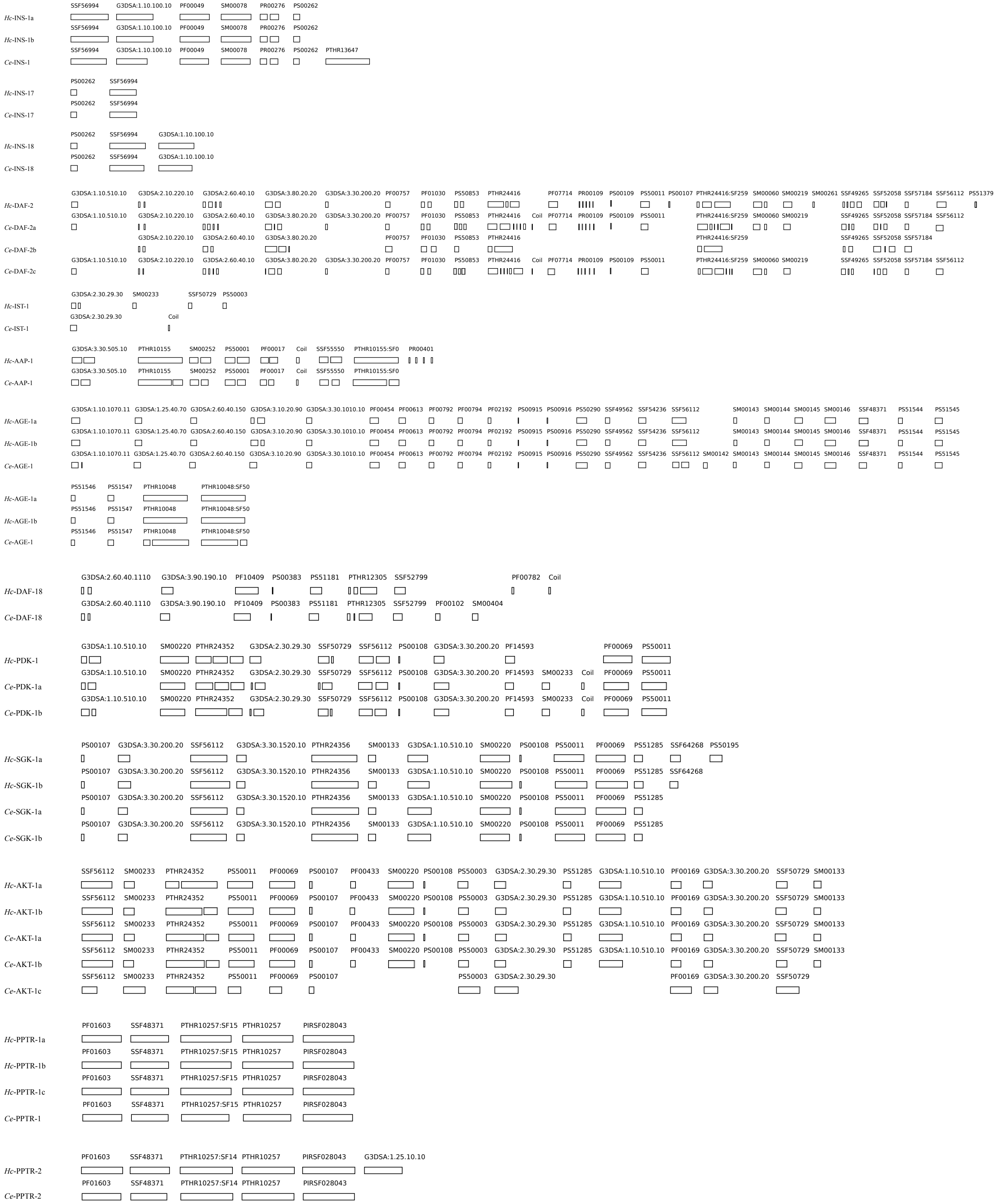

Additional file 1: Figure S1 ctd.

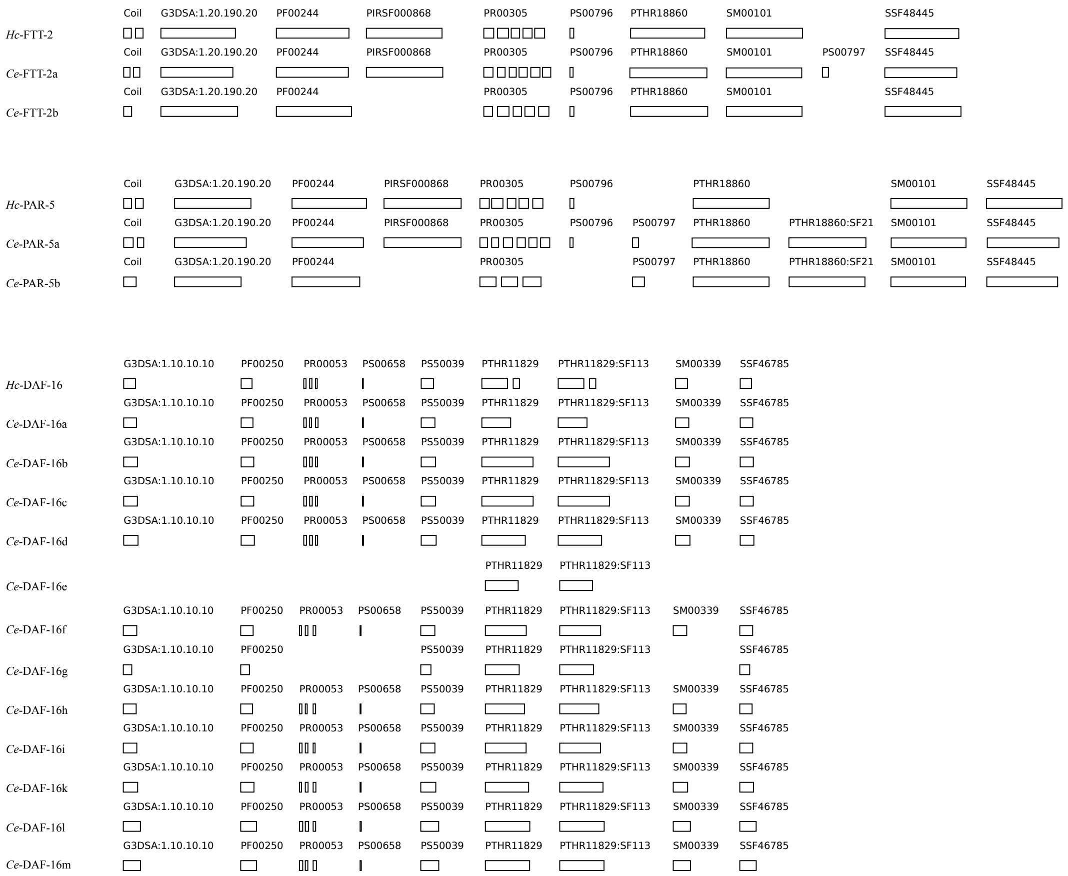

Additional file 1: Figure S1 ctd.

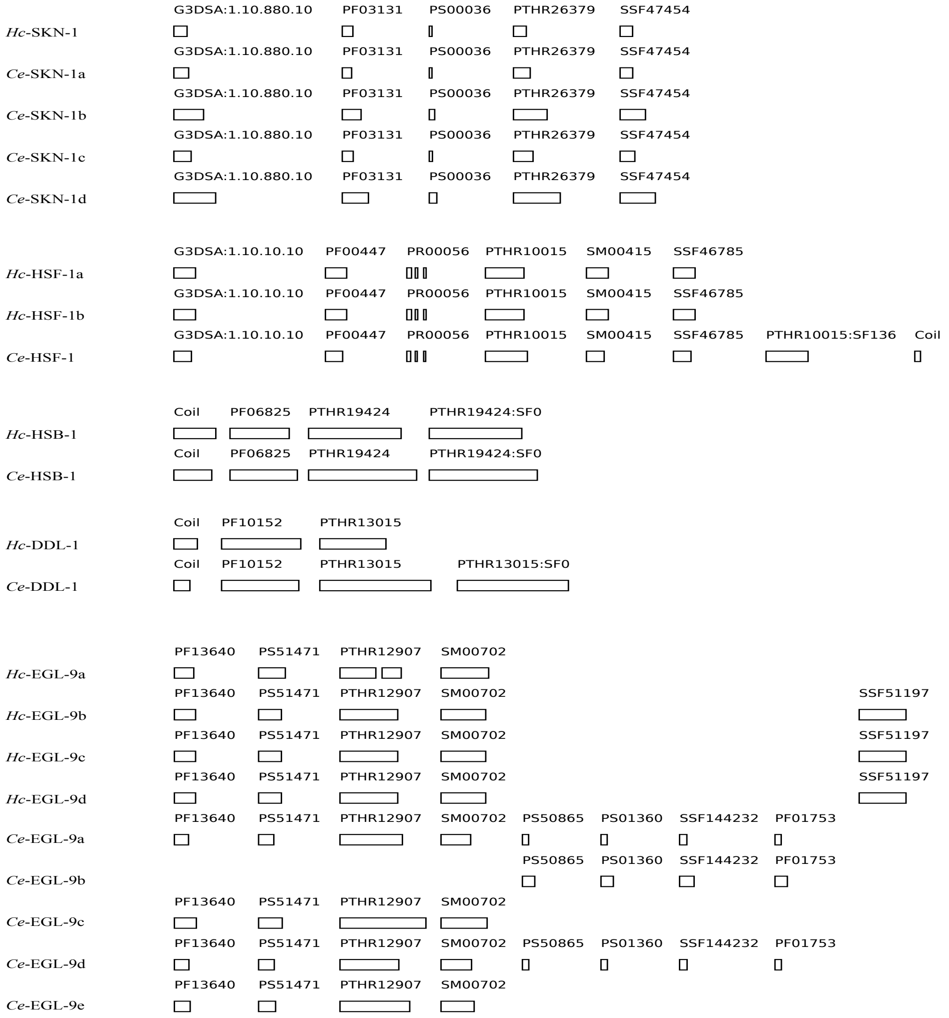

Supplement: Additional file 1: Figure S1. — A schematic representation of functional domains and motifs as predicted by InterProScan of members of the insulin/insulin-like growth factor 1 (IGF1)-like signalling (IIS) pathway of Haemonchus contortus inferred from full-length transcripts and their Caenorhabditis elegans homologs. (PDF 216 kb) [file 13071_2016_1341_MOESM1_ESM.pdf]
